# Organosilica nanoparticle-reinforced resin-based dental composites: synthesis, characterisation, and evaluation of physicochemical properties

**DOI:** 10.1039/d6ra01974a

**Published:** 2026-04-14

**Authors:** Mohammed A. Al-Khafaji, Ali O. Imarah, Athmar A. Kadhim, Mudher K. Mohammed, Hayder O. Hashim, Judith Mihály, Zoltán Varga

**Affiliations:** a Department of Basic Science, College of Dentistry, University of Babylon Hilla Iraq phar.mohammed.a.karam@uobabylon.edu.iq moh_chem_84@yahoo.com; b Department of Chemical Engineering, College of Engineering, University of Babylon Hilla Iraq; c College of Kufa, Al-Furat Al-Awsat Technical University Najaf Iraq; d Department of Pharmacy, Al-Manara College of Medical Science Amarah Iraq; e Department of Clinical Laboratory Sciences, College of Pharmacy, University of Babylon Hilla Iraq; f Institute of Materials and Environmental Chemistry, HUN-REN Research Centre for Natural Sciences Budapest Hungary; g Department of Chemistry, Eszterházy Károly Catholic University Eger Hungary; h Department of Physical Chemistry and Materials Science, Faculty of Chemical Technology and Biotechnology, Budapest University of Technology and Economics Budapest Hungary

## Abstract

Dental nanocomposites have recently gained significant attention in the scientific community due to their ability to improve the aesthetic outcomes of dental restorations, thereby improving appearance. Despite extensive research efforts, developing dental nanocomposites that meet both mechanical and aesthetic standards remains a challenging issue. Although nanoparticles are effective at enhancing dental nanocomposite materials, they also have drawbacks. The higher surface-to-volume ratio of silica particles, combined with their hydrophilic nature, increases water absorption and can cause the resin to dry, leading to nano- or micro-leakage. The study investigates the effect of a hydrophobic organo-silica shell framework on nanocomposite performance and the mechanical properties of a dental filler-based nanocomposite. Inorganic SiO_2_ nanoparticles were coated with 1,2-bis(triethoxysilyl)ethane (BTEE) to improve hydrophobicity and promote liquid-phase mixing. Core-shell organosilica nanoparticles (csOS NPs) and solid silica core nanoparticles (sSiO_2_ NPs) were conjugated with 3-(trimethoxysilyl)propyl methacrylate (γ-MPS), creating csOS-MA and sSiO_2_-MA nanoparticles with methyl methacrylate (MA) groups for better filler-resin binding. Characterization techniques included scanning electron microscopy (SEM), transmission electron microscopy (TEM), dynamic light scattering (DLS), and Fourier transform infrared spectroscopy (FTIR). Single-weight amounts of sSiO_2_-MA and csOS-MA nanoparticles were used as fillers in composites tested for compressive strength (CS), modulus, and flexural strength (FS), polymerisation shrinkage (PS), and degree of conversion (DC). The findings revealed that the organosilica shell has a significant impact on the mechanical characteristics and DC of the nanocomposite. The study examined the water sorption (WSP) and water solubility (WSL) of nanoparticles prior to and following surface modification. The findings indicated that the addition of an organosilica shell significantly reduced both WSP and WSL while also improving the overall physical properties of the nanoparticles.The findings show that incorporating an organosilica shell on the surface of an inorganic silica core significantly enhances the design of dental restorative materials for nanocomposite filling restorations.

## Abstract 2

Dental nanocomposites have recently gained significant attention in the scientific community due to their ability to improve the aesthetic outcomes of dental restorations, thereby improving appearance.

## Graphical abstract

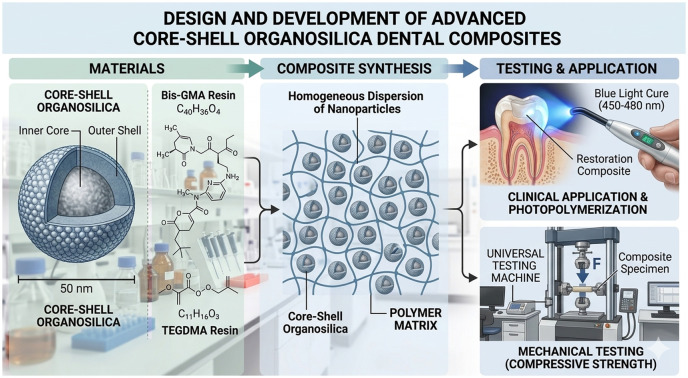

## 1. Introduction

Dental nanocomposite materials are constructed of inorganic filler particles incorporated in a polymer resin matrix that polymerises upon application. Polymer composites are popular due to their advantageous properties such as nontoxicity, biocompatibility, aesthetic appeal, and antibacterial activity.^1^ Among numerous polymers, including urethane dimethacrylate (UDMA), bisphenol A-glycidyl methacrylate (Bis-GMA) is commonly regarded as the preferred dental polymer. Its high viscosity, low volatility, and limited polymerisation shrinkage make it ideal for both anterior and posterior dental restorations. In contrast, urethane methacrylate was chosen for its higher hardness and lower viscosity. Issues arise when Bis-GMA is used as a resin due to its high viscosity. To address this, diluents such as triethylene glycol dimethacrylate (TEGDMA) and 2-hydroxyethyl methacrylate (HEMA) are incorporated to reduce the viscosity in dental composites.^2–4^ Similarly, inorganic fillers can be utilised to enhance the physical and performance characteristics of composite resins.^5,6^ Numerous efforts have been dedicated to developing inorganic fillers that deliver superior performance and ensure long-term clinical durability. These advancements focus on incorporating antibacterial properties, minimising polymerisation shrinkage, enhancing wear resistance, and achieving optimal mechanical properties, including flexural strength, compressive strength, hardness, flexural modulus, diametral tensile strength, fracture toughness, and elastic modulus.^1,7^ A variety of functionalized fillers can be introduced to achieve this. Typically, powdered silica, hydroxyapatite, zirconium dioxide, and barium glass are used as inorganic fillers in composite resins.^8–10^ As a result, current research is frequently focused on creating and enhancing fillers, including filler type, size (which develops from macrohybrid to microfill and finally nanofill), and shape.^7,8,11^

Including nanoparticles in dental composite materials improves mechanical, chemical, and optical properties, including better filler dispersion in the matrix.^12,13^ Among several compounds that are applied as inorganic fillers like apatite, zeolite and phosphate, silica nanoparticles are more promising due to their porous structure and bio-compatibility represented by low toxicity rates and low density.^14,15^ Karabela *et al.*, for example, investigated how nanosilica particles with sizes ranging from 7 to 40 nm impact the physico-mechanical characteristics of composite resins. The effects of 3-methacryloxypropyltrimethoxysilane, a silane coupling agent, were also investigated. The results revealed that, independent of filler particle size, the MPS% on a silica surface increases with decreasing silica particle size. Composite conversion with sorbed water improves flexural strength and modulus.^16^

However, as noted, the nanoparticles added to the dental composite improve its physicochemical characteristics but still have limitations. The greater surface-to-volume ratio of the nanoparticles may cause increased water sorption, which could damage the resin–matrix interface.^17^ Another disadvantage of employing nanoparticles as fillers is that, when added to high-viscosity resin monomers, their enormous surface area leads to low filler loading and poor particle wetting. On the other hand, as the agglomerate expanded, its surface area decreased. The water sorption resulted in a more wetting composite. The surface charge of nanoparticles not only results in aggregated forms but also stops them from spreading throughout the matrix phase. Maintaining proper particle dispersion causes the composite to have many weak points, which eventually causes the dental restoration to fail.^18^

In addition to fillers, an adhesive agent (often silane) was utilised to bind the fillers to the resin matrix in the composite.^19^ In adhesive dentistry, the shift from classical salinisation to current intermolecular cross-linking methodology has been revolutionised. The addition of a silane coupling agent to the filler surface of composites is expected to increase cohesive strength *via* chemical interaction. This process is accomplished by the condensation of silanol functional groups with pre-hydrolysed silane and hydroxyl groups on the surface of silica nanoparticles.^18,20^ Water absorbed by the composites may break up the silane agent binding.^21,22^ Based on this, developing a resin composite that meets both the aesthetic and mechanical requirements for anterior and posterior restorations represented a challenge.^23^ In 2006 Matinlinna *et al.* were the first to apply a dipodal silane, namely 1,2-bis(triethoxysilyl)ethane (BTEE), as a solution to the hydrolytic instability commonly associated with traditional monopodal coupling agents.^24^ Afterwards, in their subsequent 2008 investigation, they also proved the influence of silane dilution; the maximum shearing bond strength and water resistance after heat treatment was observed at 1.0% BTEE formulation (1.0 vol% MPS + 1.0 vol% BTEE).^25^ This sort of research culminated in a novel experimental design by Matinlinna *et al.* in 2010, addressing silane applications in orthodontics, especially to increase the adhesion between dental resin and prepared titanium using the dipodal structure of BTEE. The focus is on developing a strong siloxane network on silicatized surfaces those functions as a coupling agent.^26^

The research assesses shear bond strength and the clinical behaviour of the BTEE-based system, measuring its influence on bond stability and failure rates resulting from its molecular design. It was found that the utilisation of specialised silane mixtures (*e.g.* 3-acryloxypropyltrimethoxysilane with BTEE) has the potential to maximise shear bond strength and durability and may lead to significantly enhanced bonding performance.^27^ It was also discovered that the modified silane layer containing BTEE may limit fungal colonisation, providing a dual benefit of enhanced structural bonding and increased biological resistance to peri-implant infections.^28^ To the best of our knowledge, the insertion of 1,2-bis(triethoxysilyl)ethane (BTEE) modified shell-modified SiO_2_ nanoparticles into dental composite systems has yet to be investigated.

The improvement of the filler phase in this work is one of the primary variables used to develop the resin-based dental nanocomposite material. This study investigated the impact of an organosilica shell framework on the surface of silica core nanoparticles, which created inorganic–organic silica core–shell nanoparticles (csOS NPs) reinforced with γ-MPS groups on some of the physicochemical properties of light-cured nanocomposites. The resin nanocomposite included a Bis-GMA/TEGDMA (49.5/49.5 wt/wt) resin matrix, a co-initiator of 0.8% EDMAB, and an initiator of 0.2% CQ. To accomplish this, silica core nanoparticles were created using hydrolysis and condensation. A similar method was used to coat the sSiO_2_ core nanoparticles with an organosilica shell. Finally, the post-grafting approach was employed to embed γ-MPS groups on the surface. All the prepared samples were examined utilising SEM, TEM, FTIR, and DLS techniques. The contact angle was measured both before and after surface coating in order to assess the hydrophilicity of the produced samples. To investigate the effects of the organosilica shell's hydrophobicity on the physicochemical characteristics of dental nanocomposites. Single weights of sSiO_2_-MA NPs and csOS-MA NPs were added to the nanocomposites. The degree of conversion (DC), polymerisation shrinkage (PS), flexural strength (FS), modulus, and compressive strength (CS) were assessed in connection with csOS-MA NPs. Additionally, water sorption (WSP) and polymerisation shrinkage (PS) were measured and compared to SiO_2_-MA nanocomposites with the same filler weight fractions. Due to its porous structure and adsorption properties. Nanoparticles of silica dioxide possess extremely high surface activity and adsorb various ions and molecules.

## 2. Materials and methods

### 2.1 Experimental materials

Tetraethyl orthosilicate (TEOS, 99% (GC)), triethylene glycol dimethacrylate (TEGDMA, 95%), 1,2-bis(triethoxysilyl)ethane (BTEE, 96%), 3-(trimethoxysilyl)propyl methacrylate (γ-MPS, 98%), 2,2-Bis[*p*-(2′-hydroxy-3′-methacryloxypropoxy)phenylene] propane (Bis-GMA, 99%) were all provided by SIGMA-ALDRICH CHEMIE GmbH (Steinheim, Germany). The co-initiator ethyl-4-dimethylaminobenzoate (4-EDMAB, 99%), and initiator camphorquinone (CQ, 98%) both were provided from J&K Scientific GmbH (Pforzheim, Germany). Ethyl alcohol (EtOH, 99.8%), ammonium hydroxide (NH_4_OH, 25%) (v/v), hydrochloric acid (HCl, 37%), and hexadecyltrimethylammonium bromide (CTAB, 98%) were purchased from Shanghai Chemical Reagent Co., Ltd (Shanghai, China). All chemicals were used as received from the supplier without any further purification. The main text of the article should appear here with headings as appropriate.

### 2.2 Preparation of nanofiller

#### 2.2.1 Synthesis of solid silica (sSiO_2_) core particles

Solid SiO_2_ core particles with a nominal diameter of 210 nm (sSiO_2_) were produced using the Stöber method, following the approach described previously.^29^ In a 250 mL Schott Duran® borosilicate glass bottle, 6 mL of TEOS was quickly added to a mixture of 70 mL ethanol, 14 mL double-distilled water (DDW), and 3.14 mL ammonia solution (25%)while being continuously stirred at 500 rpm and 30 °C. The reaction was continued for 1 hour before the resulting sSiO_2_ particles were collected by centrifugation (10 min at 5000×*g* at room temperature in Falcon™ 50 mL conical centrifuge tubes). The final product was then vacuum-dried overnight after being washed twice in DDW and twice in absolute ethanol.

#### 2.2.2 Synthesis of inorganic–organic silica core–shell nanoparticles

A porous organosilica shell was synthesised on the surface of the pre-synthesised solid SiO_2_ core particles using a mixture of TEOS and BTEE as inorganic/organic silica precursors and CTAB as a structure-directing agent. The current study was done as detailed in our previous studies, following the optimal conditions.^29,30^ A typical method involves resuspending 100 mg of sSiO_2_ core in 20 mL DDW and sonicated until no particle aggregation was observed. Similarly, 180 mg of CTAB surfactant was dissolved in 30 mL of a 1 : 1 water–ethanol mixture containing 1.1 mL of ammonia. After 30 minutes of stirring (500 rpm), the dispersed sSiO_2_ solution was gradually added dropwise while the CTAB solution continued stirring. After that, 10 µL of TEOS was quickly added to the previously mentioned mixture, followed by 50 µL of BTEE. The reaction was continued for 6 hours before the core–shell organosilica nanoparticles (csOS NPs) were collected by centrifugation (10 min at 5000×*g* at room temperature in Falcon™ 50 mL conical centrifuge tubes). The sample was then washed twice with absolute ethanol and twice with DDW before being vacuum-dried overnight.

#### 2.2.3 Functionalization of sSiO_2_ and csOS NPs with methacrylate groups

The sSiO_2_ and csOS NPs were functionalized with methacrylate (MA) groups using 3-(Trimethoxysilyl)propyl methacrylate (γ-MPS) *via* a post-grafting technique in ethanol solution before being mixed with resin. In brief, pre-synthesised sSiO_2_ and csOS NPs were transferred into two separate Schott Duran® borosilicate glass vials and dissolved in 10 mL of DDW. To adjust the pH to 10.2, 40 mL of absolute ethanol, followed by 115 µL of 25% ammonium solution, was added to each vial. In the meantime, 260 µL of γ-MPS was added to 20 mL of absolute ethanol and stirred at room temperature for a while. The two suspensions were heated to 60 °C before quickly adding 10 mL of the γ-MPS solution to each sample. The reaction was carried out for 15 minutes, and the resultant material was collected by centrifugation (10 min at 5000×*g* at room temperature in Falcon™ 50 mL conical centrifuge tubes). The sample was washed twice with absolute ethanol and then twice with DDW to remove any unreacted γ-MPS. Finally, the final product was nominated as (sSiO_2_-MA) and (csOS-MA), and all products were examined using a Fourier Transform Infrared spectrometer (FTIR), dynamic light scattering (DLS), scanning electron microscopy (SEM), and transmission electron microscopy (TEM).

### 2.3 Preparation of dental nanocomposite

#### 2.3.1 Preparation of uncured dental nanocomposite pastes

The dental resin nanocomposite pastes were constructed out of a resin matrix and the synthesised sSiO_2_-MA and csOS-MA as fillers. The resin matrix was composed of a monomer mixture (Bis-GMA/TEGDMA, 49.5/49.5 wt/wt), camphorquinone (CQ, 0.2 wt%), and ethyl 4-dimethylaminobenzoate (4-EDMAB, 0.8 wt%) as photo initiators. After that, the appropriate amount of csOS-MA was manually mixed with the resin until the powder was thoroughly wetted with the organic matrix, and the resulting mixture was dispersed with ultrasonication for 10 minutes to achieve uniform distribution. The filler content of the resin nanocomposites was restricted to 65 wt% to guarantee paste handling qualities comparable to conventional dental nanocomposite resins. A Bis-GMA/TEGDMA matrix with sSiO_2_ nanofiller was also made with the same composition to serve as a control material.

#### 2.3.2 Light curing of dental nanocomposite pastes

The nanocomposite pastes were then placed into precisely sized plastic moulds that had been made according to the required measurements and polymerised with a curing light (Panaloux 470 nm, 550 mW cm^−2^, China) for 90 seconds on each side. Before examination, all specimens were polished using silicon carbide paper (P1200 grit). All samples were packed in dark containers before further use.

### 2.4 Measurements

#### 2.4.1 Structure, size, and morphology of synthesised fillers nanoparticles

The structure, size, and morphology of the synthesised samples were characterised using transmission electron microscopy (TEM) using a MORGAGNI 268D transmission electron microscope (FEI, The Netherlands) operated at 80 kV; Scanning electron microscopy (SEM) (S-4800, Hitachi, Japan); Dynamic light scattering (DLS) measurements were performed on a W130i instrument (AvidNano, HighWycombe, UK) using low-volume disposable cuvettes (UVette, Eppendorf Austria GmbH, Wien, Austria). Before measurements, samples were diluted tenfold with MQ water. DLS results were reported using the *Z*-average diameter (harmonic intensity averaged particle diameter) and the polydispersity index (PDI); and Fourier-Transform Infrared Spectroscopy (FT-IR) spectroscopy was applied to examine the structures of products using a Varian Scimitar 2000 FTIR spectrometer (Varian Inc., Palo Alto, CA, USA) equipped with an MCT (mercury-cadmium-telluride) detector and a single reflection ATR unit (Golden Gate, Specac Limited, Orpington, UK) with a diamond ATR element. For the measurements, 2 µL of undiluted material was air-dried onto the ATR crystal, and 128 spectral scans were recorded.

#### 2.4.2 Contact angle

The water contact angle (WCA) of SiO_2_ and csOS NPs was measured using the sessile drop technique on a Drop Shape Analyzer (DSA25, KRÜSS GmbH, Germany) using ADVANCE software. A 5 µL droplet of deionised water was deposited on the compressed sample surface, and the WCA was measured during 10 seconds for each surface. To guarantee repeatability, three samples from each sample were evaluated in three separate locations.

#### 2.4.3 Mechanical properties of dental nanocomposites

Dental nanocomposites containing csOS-MA were evaluated for flexural strength (FS), flexural modulus (FM), and compressive strength (CS) using a Universal Testing Machine (model WDW-56, China), the rectangular specimens (25 mm × 2 mm × 2 mm, *n* = 3) were made from nanocomposite paste and polymerised for 90 seconds using an LED curing machine.^31^ A three-point bending test with a 20 mm span and a cross-head speed of 0.75 mm min^−1^ was used to quantify the polymerised specimens, immersed in distilled water at 37 °C for 24 h, according to ISO 4049-2009.^32^ After drying the specimens with tissue paper, flexural strength (FS) and flexural modulus (FM) were measured using a UTM in accordance with the following equations:1
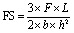
2
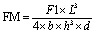
where *F* stands for load at fracture, *L* for span length, *b* for width, *d* for thickness, and *h* is the height of the test specimen (mm). *F*1 is the maximum load (N) at the straight-line part, and *d* is the deflection (mm) at load *F*1. The flexural strength was determined. To determine Compressive Strength (CS), cylindrical specimens (*ϕ* 4 mm × 6 mm, *n* = 6) were manufactured and analysed at a cross-head speed of 0.8 mm min^−1^.

#### 2.4.4 Degree of conversion

An FTIR spectrometer (Varian Inc., Palo Alto, CA, USA) with an MCT (mercury–cadmium–telluride) detector and a single reflection ATR unit (Golden Gate, Specac Limited, Orpington, UK) was used to investigate the degree of conversion (DC) of experimental dental nanocomposites. The spectral range was maintained at 4000–400 cm^−1^, with 64 scans and 4 cm^−1^ resolutions for analysis. Each nanocomposite specimen's FT-IR spectra were acquired both before and after curing. The DC of each dental resin nanocomposite was determined by comparing the absorbance intensities of aliphatic C=C bonds (1638 cm^−1^) to an internal standard of aromatic C=C bonds (1608 cm^−1^). Each nanocomposite sample was tested according to three separate tests. The DC (%) was determined using the formula below:3
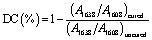
where *R* = band height at 1638 cm^−1^/band height at 1608 cm^−1^.

#### 2.4.5 Polymerization shrinkage

The polymerisation shrinkage (PS) of dental nanocomposites following polymerisation was determined using Archimedes principle.^33^ Three uncured samples for each nanocomposite were made using the above-mentioned method and weighed in both air and water, using an analytical balance (SartoriusTM CP224S, USA). In addition, samples cured for 90 seconds were weighed in both air and water. The density of the uncured and cured nanocomposites, both before and after curing, was evaluated. The polymerisation shrinkage of the nanocomposites was computed using equation.4
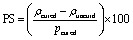
where uncured is the density of the uncured sample (g cm^−3^), cured is the density of the cured sample (g cm^−3^).

#### 2.4.6 Water sorption and solubility

The water sorption test of the SiO_2_-MA and csOS-MA filler-based nanocomposites with two specimens of each type was tested. Upon polymerisation, the samples were then placed in a preconditioning oven set at 37 °C. After 48 hours, samples were weighed on a digital scale (precision 0.01 g) to establish a consistent mass (within ± 0.1 mg) and recorded as (*m*_1_). After, the sample was immersed in 100 mL of distilled water at 37 ± 1 °C. The samples were submerged in 37 °C distilled water for 21 days. The samples were blotted dry on filter paper to remove excess water before being weighed at 1, 7, and 21 days (*m*_2_) and restored to the water. Using the same technique, water intake was measured until there was no significant change in weight, indicating equilibrium (*m*_3_). The samples were subsequently delivered to a 37 °C drying oven, and the process described above was repeated during desorption. Once the sample weight reached equilibrium, the samples were returned to the water, and a second absorption cycle was recorded with the same measurement intervals.^34^ The water absorption and water solubility of each sample were calculated separately with the following equations;5
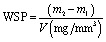
6
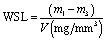
where WSP and WSL represent water sorption and water solubility, respectively, *m*_1_, *m*_2_, and *m*_3_ represent different mass measurements, and *V* represents the volume of the sample.

#### 2.4.7 Statistical analysis

Statistical analysis was performed to identify significant differences between groups using the independent samples *t*-test. For the mechanical attribute data, results were expressed as mean ± standard deviation (SD). The sample sizes were *n* = 3 for all under investigation parameters, and *n* = 6 for compressive strength. Data processing and graphical representations were conducted using IBM SPSS Statistics software (Version 26.0; IBM Corp., Armonk, NY, USA).

## 3. Results and discussion

### 3.1 Structure and morphology characterisation

**Fig. 1:**
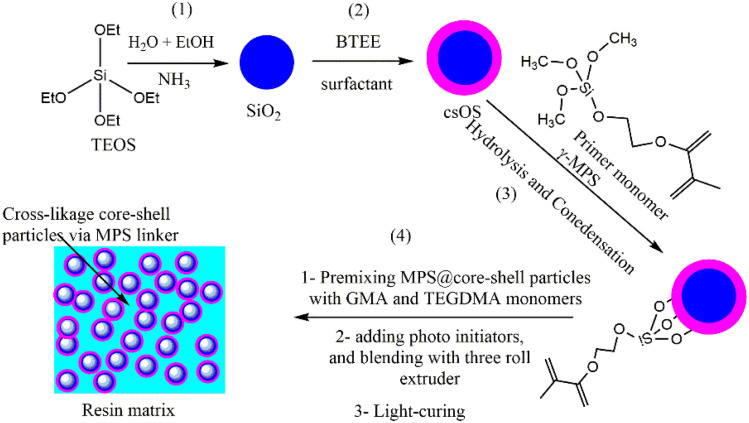
Representation of the preparation procedure of sSiO_2_ NPs, csOS and surface modification with MA groups.

Inorganic–organic silica core–shell nanoparticles (csOS NPs) were produced by creating a shell framework using 1,2-bis(triethoxysilyl)ethane (BTEE) as an organo-silica source on the surface of pre-synthesised solid silica cores. This was followed by grafting the surface of NPs with methacrylate (MA) groups using 3-(trimethoxysilyl)propyl methacrylate (γ-MPS). The preparation process is shown in Fig. 1.

**Fig. 2:**
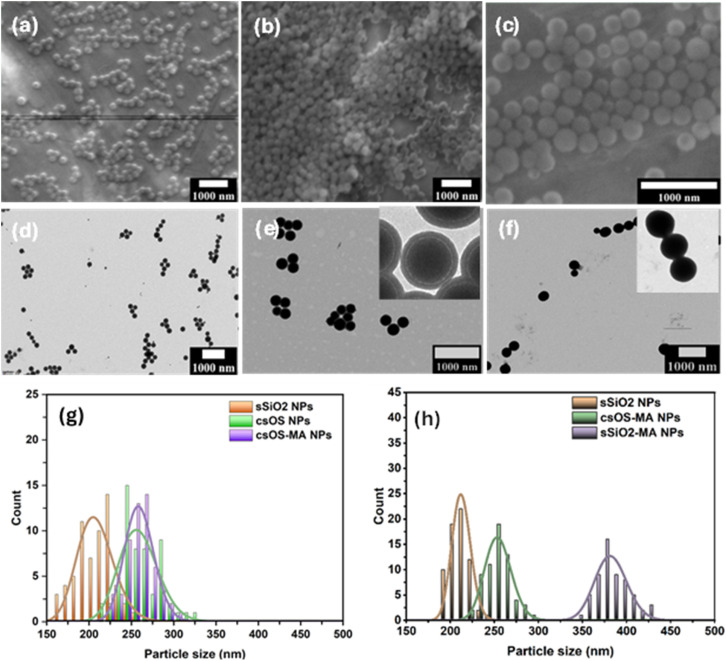
Displays SEM images of sSiO_2_ NPs (a), csOS NPs (b), and csOS-MA NPs (c), as well as TEM images of sSiO_2_ NPs (d), csOS-MA NPs (e), and sSiO_2_-MA NPs (f). The histogram depicts the particles' outer diameters as determined by SEM (g) and TEM (h).

This is achieved by first creating monodisperse sSiO_2_ NPs using the Stöber technique, with a diameter of ∼210 nm and a spherical shape, as observed by SEM and TEM (Fig. 2a and d). The findings showed that the particle size of the silica (sSiO_2_) core was approximately consistent, as evidenced by the data obtained from SEM and TEM, as illustrated in Fig. 2(a and d). After successful formation of sSiO_2_ NPs, csOS NPs were created by hydrolysis and condensation of BTEE on the surface of sSiO_2_ NPs, with a percentage of CTAB surfactant as a pore formation agent, as shown in step 2 (Fig. 1). The particle size and surface shape of csOS NPs, as determined by SEM and TEM measurements, are also shown in Fig. 2. The finding suggested that the particle size of csOS NPs increased to about 256 nm in diameter after shell formation as observed by SEM (Fig. 2b). The surface modification of csOS NPs with MA groups has been carried out in an ethanol solution using γ-MPS to improve the adhesion force between the resin matrix and filler. The SEM examination of these particles revealed a slight increase in size, with an average size of around 259 nm (Fig. 2c). The particle size of synthesised csOS NPs after surface modification, denoted as (csOS-MA), was further confirmed by TEM examinations, which indicated a shell thickness of around 23 nm.The results demonstrated that the created shell formwork remained intact and homogeneous spherical shapes with no sign of particle aggregation. Furthermore, the low density of this shell is further proof of its formation, shown in the inset image of TEM measurement (Fig. 2e). To evaluate the size distribution of synthetic samples, at least 50 particles were analysed using ImageJ. Gaussian fitting was then used to calculate the average particle sizes; Fig. 2(g and h) display the size distribution of the particles measured by SEM and TEM, respectively.

The results were listed in Table 1. The hydrodynamic dimensions and PDIs of the synthesised NPs as determined by dynamic light scattering (DLS) are also displayed in Table 1. The results revealed that the hydrodynamic diameter of these NPs is slightly greater than that assessed by SEM and TEM for the same size. This is explained by the hydrodynamic diameter measured by DLS, which reflects the hydrated layer on the particle's outer surface. The PDI values of the measured particles were similarly low, indicating that the particles are monodisperse and show no evidence of aggregation, consistent with the results from SEM and TEM observations. Additionally, sSiO_2_ NPs were surface modified with MA groups using the same technique used to prepare csOS-MA NPs. These NPs were employed as a reference material to examine the water sorption and bonding strength of csOS-MA NPs when utilised as a filler in dental nanocomposites. The particle sizes of sSiO_2_-MA NPs, as measured by TEM and SEM, are given in Table 1 and are about 382 and 363 nm, respectively. In contrast to csOS NPs, it has been noted that sSiO_2_ particles exhibit a significant increase in particle size following silanation with MA groups. The hydrophilic characteristics of inorganic sSiO_2_ NPs may explain this, as they increase the particles' ability to hydrolyse silane groups, forming multiple layers on their surface and causing a significant shift in particle size. On the other hand, the hydrophobicity of the organic groups on the surface of csOS NPs reduces cocondensation of silane groups, resulting in a thinner, more uniform layer. This is indicated by a slight increase in particle size following surface modification.^35,36^ The successful fabrication of organosilica shells was further proved when TEM examinations of sSiO_2_-MA NPs (Fig. 2f) revealed a constant particle density, but TEM observations of csOS-MA NPs (Fig. 2e) revealed a low-density shell structure caused by organosilica shell creation. The SEM measurements revealed spherical particles, as shown in Fig. S1. According to the DLS data given in Table 1, the particle size was around 322 nm, with a polydispersity index (PDI) of 0.11.

**Table 1:** Displays the hydrodynamic diameter (Dh) and polydispersity index (PDI) measured by DLS and mean particle size and standard deviation (SD) values from SEM and TEM for the particles under investigation

Sample	DLS		SEM		TEM	
	Dh (nm)	PDI	D (nm)	SD (nm)	D (nm)	SD (nm)
sSiO_2_ NPs	221.49	0.024	208.8	21.1	212.5	10.6
csOS NPs	—	—	256.1	23.3	—	—
csOS-MA NPs	292.23	0.083	259.7	17.4	254.4	15.3
sSiO_2_-MA NPs	322.16	0.117	363.3	14.3	382.5	18.4

### 3.2 Chemical structures

**Fig. 3:**
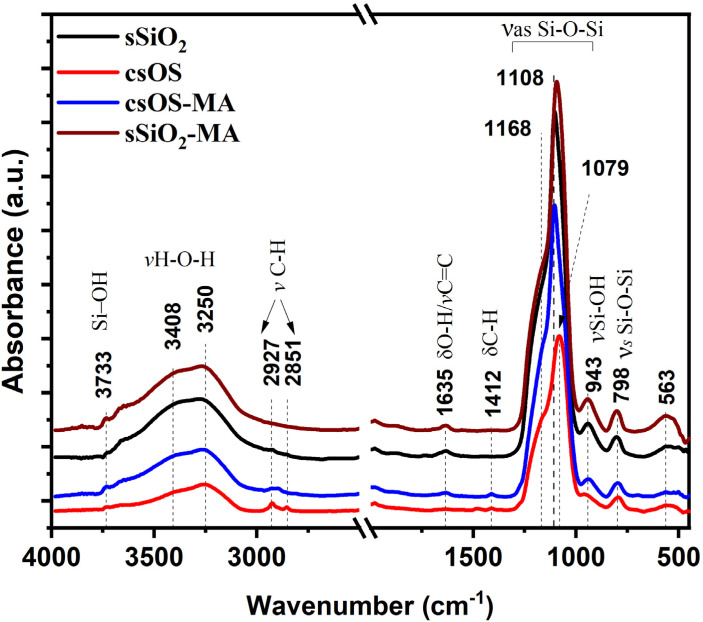
FT-IR spectra of sSiO_2_ core (black line) and csOS (red line) before and after surface modification with methacrylate groups csOS-MA (blue line) and sSiO_2_-MA (brown line). For better visualisation, the spectra are moved vertically and normalised to the highest Si–O–Si band intensity.

The chemical structures of the sSiO_2_ and csOS NPs before and after surface modifications with MA groups were examined using FT-IR, and the results are shown in Fig. 3. All synthesised samples exhibited absorption bands at 3733 cm^−1^, 3409 cm^−1^, 1108 cm^−1^, 943 cm^−1^, and 798 cm^−1^. The broad absorption bands at 3733 cm^−1^ and 3409 cm^−1^ correspond to the stretching vibrations of the O–H bond of Si–OH and water. The distinctive absorption band of the Si–O–Si bond is comprised of two peaks at about 1108 cm^−1^ and 798 cm^−1^, attributed to the symmetric and asymmetric vibrations, respectively. After the fabrication of the organosilica shell, it was found that the absorption band at 1108 cm^−1^, which was attributed to the asymmetric vibration of the regular tetrahedral structure of Si–O–Si in the sSiO_2_ spectrum, was shifted to a lower frequency and appeared at 1079 cm^−1^. This could be attributed to the deformation of the SiO_4_tetrahedron structure, which was briefly described in our previous study.^29,35^ It has also been demonstrated that surface modification of sSiO_2_ NPs and csOS NPs with MA further shifts the Si–O–Si absorption bands at 1108 cm^−1^ of the sSiO_2_ spectra to lower frequencies, reaching 1099 cm^−1^ and 1097 cm^−1^, respectively. The Si–OH bond vibration is represented by the absorption band at 943 cm^−1^. The shoulder, which was identified at about 1192 cm^−1^, was attributed to a split of longitudinal and transverse optical stretching motion.^37,38^ In comparison to the sSiO_2_ spectrum, when BTEE precursor and/or γ-MPA were fabricated on the surface of sSiO_2_, new peaks appeared at around 2964 cm^−1^, 2851 cm^−1^, and 1412 cm^−1^. The peaks at 2964 cm^−1^ and 2851 cm^−1^ represent the stretching vibration of the C–H group, while the band at 1412 cm^−1^ represented C–H deformation vibration, indicating successful surface modification.^29,30^ The water deformation band appears at around 1635 cm^−1^.^39^ As shown in Fig. 3 (red and blue lines), the hydrophobic effect of the organosilica shell, which prevents the accumulation of water molecules on the surface while also reducing the number of silanol hydroxyl groups on the surface, could explain the absence of this band in csOS spectra and its decreased intensity in csOS-MA spectra. The decreasing peak at 943 cm^−1^, ascribed to the Si–OH bond vibration, supports these predictions. Furthermore, when the csOS spectrum is compared to the SiO_2_-MA and csOS-MA spectra, as depicted in Fig. 3 (blue and brown lines), a new distinct peak at 1635 cm^−1^ is observed. This peak is attributed to the contraction vibration of C=C in γ-MPA and could be covered by the O–H vibration peak. This implies that γ-PMA was effectively grafted onto the csOS NPs and sSiO_2_ surfaces.^40^ These results demonstrate the effective synthesis of sSiO_2_ and csOS NPs modified with MA groups, which are consistent with TEM and SEM investigations.

#### 3.2.1 Nanoparticles surface hydrophobicity

The hydrophobicity of the csOS NPs was also assessed in contrast to the sSiO_2_. Fig. S2 depicts the contact angle measurements of the tested sample. The WCA of SiO_2_ NPs was around 39.16°, indicating a highly hydrophilic nature and fast droplet dispersal. The csOS NPs demonstrated a considerable rise in WCA to about 112.76°, indicating a shift toward hydrophobic behaviour due to the incorporation of non-polar ethylene bridges into the silica.

The framework bridges reduce the density of hydrophilic silanol groups. The calculated *p*-value was *p* < 0.0001, which falls below the critical threshold of *p* < 0.05. This finding is consistent with that of letreture, which shows that the insertion of organosilica shell exhibits exceptional hydrolytic stability, being up to 10 000 times more resistant to water degradation than conventional silanes, resulting in consistent wettability in aqueous environments.^41,42^ This notion was also tested in our prior work, which found that, although ordinary silica is very hydrophilic and unstable in aqueous solution at high temperatures, the BTEE organosilica shell has moderate hydrophobicity and excellent water stability, even at high pH levels.^29^

### 3.3 Resin-based dental nanocomposites study

#### 3.3.1 Organic bond properties

**Fig. 4:**
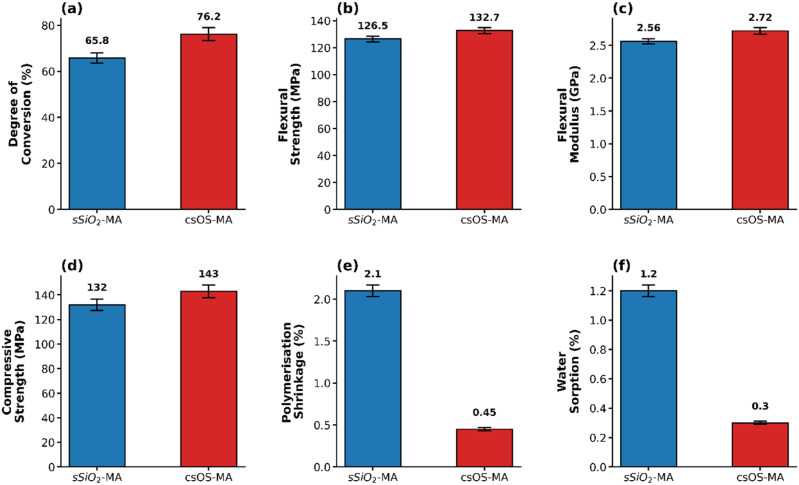
Presents a comparative analysis of csOS-based dental composites *versus* SiO_2_-based dental composites across several parameters (a) degree of conversion, (b) flexural strength, (c) flexural modulus, (d) compressive strength, (e) polymerisation shrinkage, and (f) water sorption. Bars represent mean values, and error bars indicate standard deviation (±SD).

The degree of conversion (DC) of nanocomposite materials, namely SiO_2_-MA and csOS-MA nanoparticles, was determined using eqn (3), which was explained in section 2.4.3 of the study. The results of this calculation, which relied on the spectral peaks apparent in Fig. S3, are listed in Table 2. The CSOS-MA NPs-based nanocomposite has shown an improvement in DC over the SiO_2_-MA NPs-based nanocomposite, as shown in Fig. 4a. Hence, the results of the DC of the nanocomposite using cs-OS-MA as a filler have shown that the increase in DC by about 10.5% is about 76.8% instead of 65.8% in the nanocomposite that used sSiO_2_-MA as a filler. This could be attributed to the surface porosity after organosilica shell formation, which enhances interference from methacrylate groups, which are more widely distributed on the surface. Additionally, the presence of a porous organosilica shell structure on the inorganic silica surface increases light scattering, which may result in a greater DC (*p* < 0.05).^43^ The high degree of conversion (DC) can also be attributed to constructive scattering resulting from the introduction of ethylene bridges (CH_2_–CH_2_). This modification promotes a homogeneous distribution of the particles and reduces the refractive index mismatch (Δ*n*) between the filler and the resin matrix.^30^ According to Mie theory, this intermediate contrast leads to predominant forward scattering, which extends photon optical paths and enhances the rate of photo-initiator activation.^44^

**Table 2:** Presents the physicochemical properties of the synthesised nanocomposite, including degree of conversion, flexural strength, flexural modulus, compressive strength, polymerisation shrinkage, and water sorption. Data are presented as mean (±SD)^a^

Sample	Degree of conversion (%)	Flexural strength (MPa)	Flexural modulus (GPa)	Compressive strength (MPa)	Polymerisation shrinkage (%)	Water sorption (%)
sSiO_2_-MA	65.8 (2.2)	126.5 (2.15)	2.56 (0.04)	132 (4.6)	2.1 (0.07)	1.2 (0.04)
csOS-MA	76.2 (2.8)	132.7 (2.22)	2.72 (0.05)	143 (5.2)	0.45 (0.02)	0.3 (0.01)
*P*-value	0.012	0.041	0.033	0.003	<0.001	<0.001

a Note: statistical analysis was performed using the independent samples *t*-test. All obtained *P*-values were found to be (*p* < 0.05).

The shell, characterised by its nanoporosity, creates internal surfaces that effectively trap photons. Additionally, the particles function as ‘nano-optical fibres’, channelling light both laterally and downward. This interaction facilitates efficient energy utilisation from the incident light throughout the entire functional thickness. As a result, appropriately controlled scattering by these hybrid fillers enhances both optical performance and polymerisation efficiency.

#### 3.3.2 Mechanical properties

The mechanical properties of a CSOS-MA filler-based nanocomposite were compared to those of a SiO_2_-MA filler-based nanocomposite to determine how a dental restorative would perform in the long term. For temporary dental nanocomposites to endure chewing stresses, they must have sufficient flexural strength. Compared to the SiO_2_-MA filler-based nanocomposite, the flexural strength of the csOS-MA filler-based nanocomposite was greater (Table 2). The flexural strength of the SiO_2_-MA filler-based nanocomposite was around 126.5, whereas that of the csOS-MA filler-based nanocomposite was about 132.7. For the same concentration, it was discovered that the csOS-MA filler-based nanocomposite was around 5% larger than the SiO_2_-MA filler-based nanocomposite. This could be because the porosity of the shell framework causes more MA groups to disperse across the surface, increasing the surface area. It is noteworthy that the flexural strength of the samples investigated in this study readily exceeds the ISO 4049:2019 standard flexural strength, which is 50 MPa for dental materials.^45^

The flexural modulus of both under-investigation samples was also determined using the three-point bending test for nanocomposite samples, and the findings are presented in the table. The flexural modulus was increased by about 6.25% in the case of using csOS-MA as a filler instead of SiO_2_-MA. Additionally, the compression strength of dental restorations was examined and presented in Table 2. The csOS-MA NPs-based nanocomposite outperformed the SiO_2_-MA NPs-filled nanocomposite by about 8.3% in compressive strength. The compressive strength of the inorganic silica filler-based nanocomposite was around 132 MPa, whereas the organosilica filler-based nanocomposite was around 143 MPa. Employing csOS-MA NPs could enhance the compressive strength of dental restorations more effectively than SiO_2_-MA NPs, owing to their superior mechanical properties. In terms of modulus, flexural strength, and compression, the identical trend seen in Fig. 4b–d implies that the influence of the organosilica nanoparticle is similar in the changed specimens. Significant differences (*p* < 0.05) were identified across FS, FM, and CS groups, indicating significant diversity in performance. The improved qualities reported in the material, following surface fabrication with organosilica that contains ethylene bridage, are most likely due to the mechanical and structural phenomena of micro-flexibility. The Si–CH_2_–CH_2_–Si bridges of BTEE lend “organic flexibility” to the inorganic backbone, in contrast to the rigid and brittle character of inorganic silica.^46^ These bridges act as molecular-scale springs, giving the composite increased fracture toughness by allowing for minor structural deformation without breaking.^47^ The resin may produce an interpenetrating polymer network by penetrating the filler particles due to its high porosity. As a result, the resin solidifies inside the pores, improving bonding and creating “mechanical anchors”.^48^ This causes the failure mode to change from adhesive failure at the interface to cohesive strength. Additionally, the organosilica shell acts as a buffer zone between the flexible resin matrix and the rigid filler, significantly reducing interface stress and so successfully preventing macroscopic fractures in composites.^49^

#### 3.3.3 Polymerisation shrinkage

Despite previous statements, polymerisation shrinkage (PS) remains unresolved. Nonetheless, this study reveals-great progress in solving this issue. Table 2 shows how PS decreased by over 78.5% when csOS-MA NPs were employed as a filler instead of SiO_2_-MA NPs. Fig. 4e depicts the difference between the PS of a nanocomposite formed using csOS as a filler and SiO_2_. These results were compatible with the increase in DC and improvements in the physical properties of dental restorative materials after using csOS-MA instead of SiO_2_-MA as a filler. This finding supports our hypothesis that hydrophobic organosilica materials can be used as filler-based dental restorative materials, increasing longevity while reducing secondary decay. The significant role of allowing increased DS while simultaneously lowering PS is explained by the porous organosilica shell, which has a unique decoupling effect. As it has already been mentioned in Section 3.4.2, the interior voids facilitate the growth of polymer chains into the pores, which causes micro-mechanical interlocking to transfer shrinkage from a macroscopic to a microscopic level. The ethylene-bridged groups provide increased flexibility and a low Young's modulus, acting as a “chemical shock absorber” in contrast to standard SiO_2_. Such buffering dynamics minimise the bond-formation-related stress and lead to extraordinary mechanical properties for a localised shrinkage rate of 0.45%.^50,51^ The statistical analysis showed (*p* < 0.05, indicating sufficient evidence to declare the superiority in performance as well as the stress buffer effect statistically significant. This is a substantial and validated advantage of csOS-MA over silica fillers.

#### 3.3.4 Water sorption

Water sorption was calculated using the method outlined in section 2.4.5 of this study and the data displayed in Table 2. The statistical analysis revealed considerable diversity in WSP performance among the examined groups (*p* < 0.05). The results showed that water sorption was dramatically decreased by about 75% when hydrophobic organosilica was added to the surface of the hydrophilic inorganic silica core. Fig. 4f depicts the difference between the WSP of a nanocomposite formed using csOS as a filler and SiO_2_. While the water uptake for hydrophobic organic silica-based filler was around 0.3% Wt, the water uptake for inorganic silica-based filler was approximately 1.2% Wt, which is consistent with the known hydrophilic nature of Bis-GMA/TEDMA. That which is below ISO 4049's acceptable limit of 40 µg mm^−3^ for polymer-based dental restorative materials.

## 4. Conclusions

In the current study, the impact of csOS-MA NPs on reinforced dental nanocomposite materials in comparison to sSiO_2_-MA NPs has been thoroughly investigated. NPs have been effectively synthesised using the sol–gel approach and silane-coated using the post-grafting technique. A single-weight NPs-to-matrix ratio was used to create the nanocomposite resins. The study's results showed that the overall mechanical characteristics and degree of conversion were improved by using csOS-MA NPs as a filler nanocomposite rather than sSiO_2_ NPs. The independent *t*-test results showed that samples with functionalized particles possessed a significant increase in flexural modulus, flexural strength, and compressive strength. The organic–inorganic framework and ethylene bridges contribute to these enhancements by improving the bonding with the polymer matrix. This alteration resulted in a change in contact angles, shifting from approximately 39.16° (hydrophilic) to around 112.76° (hydrophobic). The presence of a porous shell structure and effective surface modification was confirmed through FTIR and TEM analyses. These modifications have enhanced the performance of the nanocomposite and maintained its structural integrity. Combining these fillers with standard Bis-GMA/TEGDMA monomers increases hardness, strength, and conversion degree while decreasing polymerisation shrinkage and water sorption. Such advancements are especially important for restorative engineering in dentistry, where superior mechanical properties and conversion rates of reinforcing materials in nanocomposite resins can increase durability. A crucial finding of the study is that organic groups reduce water absorption, thereby minimising shrinkage and enhancing the longevity of dental nanocomposites. While this work focuses on the key physicochemical features of the produced nanocomposites. However, further study is needed to thoroughly evaluate the impacts of hybrid shell thickness and nanoporosity. It is also critical to assess long-term hydrolytic stability and biocompatibility to provide clinical dependability in complex oral environments.

## Author contributions

M. A. A., A. O. I. and A. A. K. contributed to the project conceptualisation; M. A. A., A. O. I., A. A. K., and H. O. H. conceived the methodology and designed the experiments; M. A. A., M. K. M., H. O. H., and Z. V. performed the experiments and analysed the data; A. O. I. and A. A. K. conducted the statistical analysis of the experimental data. M. A. A. and Z. V. carried out the writing-original draft preparation. All authors contributed to the writing-review of the paper. All authors have read and agreed to the published version of the manuscript.

## Conflicts of interest

The authors declare no conflict of interest.

## Supplementary Material

RA-016-D6RA01974A-s001

## Data Availability

The data that support the findings of this study are available on reasonable request from the corresponding author. All the data produced and/or analyzed in the course of this study are presented within this manuscript and its accompanying supplementary information (SI) file. Supplementary information is available. See DOI: https://doi.org/10.1039/d6ra01974a.
