# Supplementary material for: Organosilica nanoparticle-reinforced resin-based dental composites: synthesis, characterisation, and evaluation of physicochemical properties
Source: RSC Adv. 2026 Apr 14;16(22):19718–28. doi: 10.1039/d6ra01974a (PMC13078368; doi:10.1039/d6ra01974a)
Supplement: RA-016-D6RA01974A-s001 [file RA-016-D6RA01974A-s001.pdf]

Supplementary Information (SI) for RSC Advances.
This journal is © The Royal Society of Chemistry 2026

Organosilica nanoparticle-reinforced resin-based dental composites: synthesis, characterisation, and evaluation of physicochemical properties

Mohammed A. Al-Khafaji, *[a] Ali O. Imarah, [b] Athmar A. Kadhim, [c] Mudher K. Mohammed, [d] Hayder O. Hashim, [e] Judith Mihály [f,g] and Zoltán Varga [f,h]

a. Department of Basic Science, college of Dentistry, university of Babylon, Hilla, Iraq. Email: phar.mohammed.a.karam@uobabylon.edu.iq, moh_chem_84@yahoo.com.

b. Department of Chemical Engineering, College of Engineering, University of Babylon, Hilla, Iraq.

c. College of Kufa, Al-Furat Al-Awsat Technical University, Najaf, Iraq.

d. Department of Pharmacy, Al-Manara College of Medical Science, Amarah, Iraq.

e. Department of Clinical Laboratory Sciences, College of Pharmacy, University of Babylon, Hilla, Iraq.

f. Institute of Materials and Environmental Chemistry, HUN-REN Research Centre for Natural Sciences, Budapest, Hungary.

g. Department of Chemistry, Eszterházy Károly Catholic University, Eger, Hungary.

h. Department of Physical Chemistry and Materials Science, Faculty of Chemical Technology and Biotechnology, Budapest University of Technology and Economics, Budapest, Hungary.

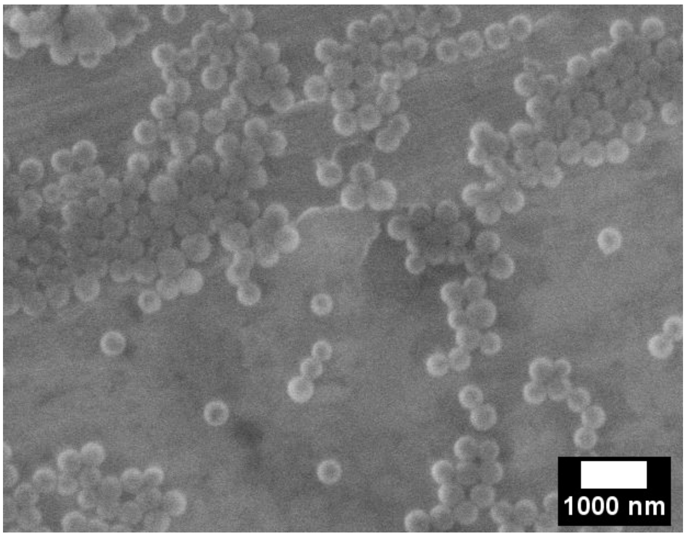

Figure S1 shows the SEM image of $sSiO_2$-MA, indicating a particle size of approximately 259.7 nm with a standard deviation of about 17 nm.

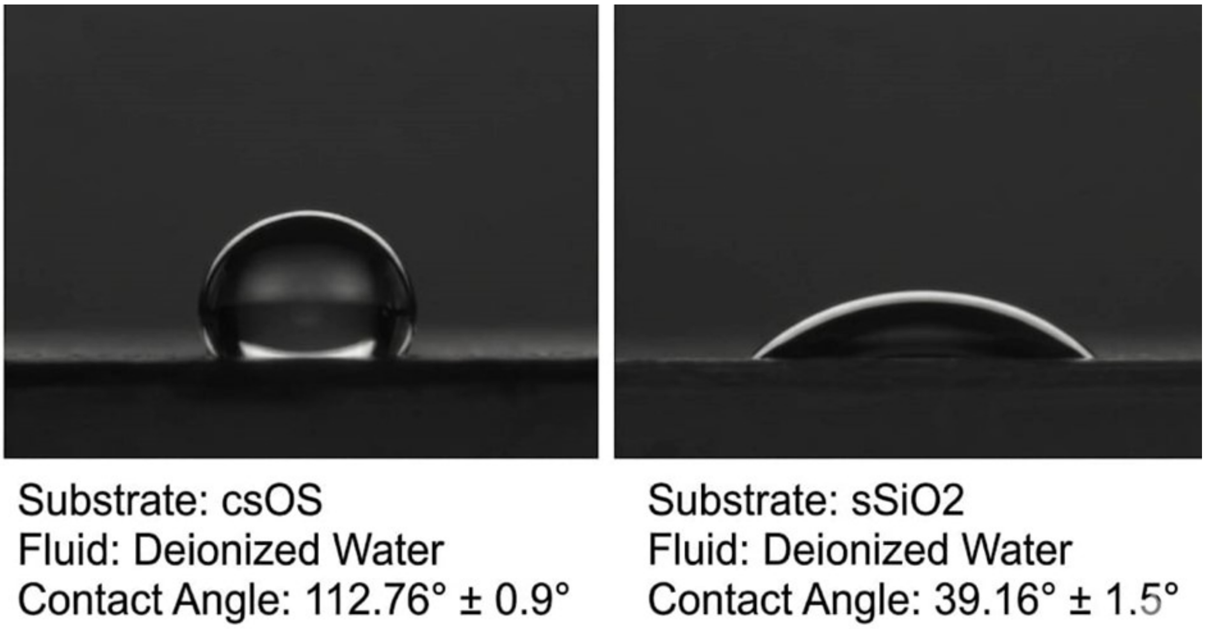

Figure S2 Shows the comparison of water contact angles on csOS and $sSiO_2$ substrates.

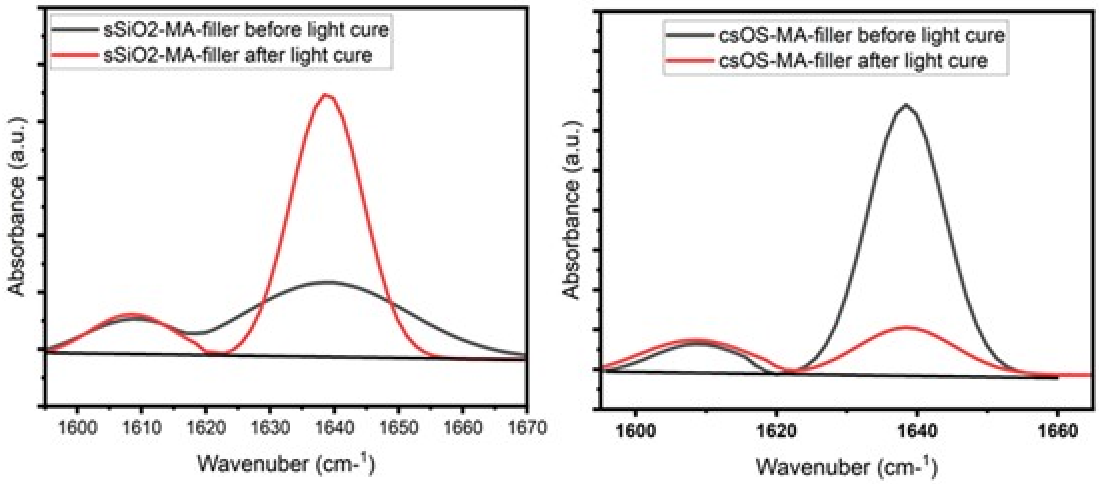

Figure S3 FTIR spectra showing the absorbance of aliphatic double bonds C=C at 1640cm^{-1} for $sSiO_2$-MA-filler and csOS-MA-filler before and after light curing.
